# Generation of a single pulmonary pressure-volume curve does not durably affect oxygenation in patients with acute respiratory distress syndrome

**DOI:** 10.1186/cc4936

**Published:** 2006-06-01

**Authors:** Antoine Roch, Jean-Marie Forel, Didier Demory, Jean-Michel Arnal, Stéphane Donati, Marc Gainnier, Laurent Papazian

**Affiliations:** 1 Service de Réanimation Médicale, Hôpitaux Sud, Marseille, France

## Abstract

**Introduction:**

It is possible that taking a static pressure-volume (PV) measurement could durably affect oxygenation and thus interfere with early evaluation of a therapeutic intervention delivered just after that measurement. The aim of the present study was to investigate the effects over time of a single static PV measurement on gas exchange and haemodynamics; the PV measurements were taken using a super syringe and by using the constant flow method in patients with acute respiratory distress syndrome.

**Method:**

We conducted a prospective, randomized and controlled interventional study in an intensive care unit. The study was conducted in 17 patients with early acute respiratory distress syndrome ventilated with a tidal volume of 6.9 ± 1.0 ml/kg, a plateau pressure of 27 ± 7 cmH_2_O and a positive end-expiratory pressure [PEEP] of 10 cmH_2_O. They were all evaluated for 1 hour after each of the following two measurements was taken and during a control period (in a randomized order): generation of a PV curve using a 2 l super syringe (PV_SS_; insufflated volume = 1824 ± 381 ml, plateau pressure = 46 ± 9 cmH_2_O); and generation of a PV curve using the constant flow method on the ventilator (PV_CF_; insufflated volume = 1120 ± 115 ml in zero end-expiratory pressure after 20 s expiratory pause, plateau pressure = 46 ± 11 cmH_2_O). The maximal airway pressure allowed during PV measurement was 60 cmH_2_O. PEEP was set to 10 cmH_2_O immediately after PV measurement. Partial arterial oxygen tension (Pao_2_), partial carbon dioxide tension (Paco_2_) and mean arterial pressure were recorded each minute.

**Results:**

PV measurement did not significantly affect Pao_2_, Paco_2_, mean arterial pressure and lung mechanics. Two patients exhibited a sustained increase in Pao_2 _by more than 20% after PV_CF _(>60 minutes). Two patients exhibited a decrease in Pao_2 _by more than 20% after PV_SS_, which was sustained in one. These latter patients had an upper inflection point identified on the PV curve. After PV_SS_, Paco_2 _increased by more than 10 mmHg in two patients and returned to baseline values after 15 minutes. One patient exhibited a decrease in mean arterial pressure by more than 10 mmHg for less than 5 minutes after PV_SS _and one patient after PV_CF_.

**Conclusion:**

Evaluation of the effects of a strategy aimed at improving oxygenation can be reliably recorded early after a single PV measurement that is not followed by a change in PEEP level. PV measurement using the constant flow method improves oxygenation in a limited number of patients.

## Introduction

The pressure-volume (PV) curve characteristics of the respiratory system are commonly evaluated in clinical and experimental studies of acute respiratory distress syndrome (ARDS). The PV measurement involves insufflating the lungs at low flow with a volume of up to 2 l using a super syringe [1] or about 1200 ml by ventilator [2], which is done in order to construct a static PV curve. The procedures required to construct PV curves may improve oxygenation because they result in alveolar recruitment. On the other hand, there are aspects of the two procedures that could result in impaired oxygenation; specifically, it is necessary to disconnect the patient from the ventilator before and after PV curve measurements with the super syringe technique, and with the constant flow method positive end-expiratory pressure (PEEP) must be removed before the PV curve measurements can be taken [3,4]. However, the potential sustained effects of PV measurement on gas exchange and haemodynamic parameters have not been investigated in patients presenting with acute respiratory distress syndrome (ARDS). This is of concern when ventilator settings (such as adjusting PEEP level) or any other intervention (for example prone positioning) are studied just after PV measurement and evaluated by blood gas analysis during the following 20–60 minutes. In these situations it is important to know how long one should to wait after PV measurement to obtain stable oxygenation parameters. The present study, conducted in ARDS patients, compared the effects over time of a single static PV measurement – using the super syringe and the constant flow method – on gas exchange.

## Materials and methods

The study was approved by our ethics committee. Seventeen consecutive patients were investigated early in the course of ARDS (<24 hour) once written informed consent had been obtained from each patient's next of kin. Patients met the following criteria: arterial oxygen tension (Pao_2_)/fractional inspired oxygen (Fio_2_) ratio of 200 or less, bilateral radiographic pulmonary infiltrates, and pulmonary artery occlusion pressure of 18 mmHg or less [5]. A computed tomography scan was performed during the preceding 12 hours to classify pulmonary infiltrates as diffuse, lobar, or patchy [6]. Patients were sedated, paralyzed and ventilated under volume control ventilation (Puritan Bennett 840; Puritan Bennett, Carlsbad, CA, USA) using the following parameters throughout the study: tidal volume at 6–7 ml/kg ideal body weight, plateau pressure (Pplat) below 35 cm H_2_O, Fio_2 _at 0.8 and PEEP at 10 cmH_2_O.

Patients were studied during three randomly assigned and successive 1-hour periods, two of which were after the following interventions one was a control period: a PV measurement performed using a 2 l super syringe (PV_SS_) and a PV measurement performed using the constant flow method (PV_CF_). PV_SS _measurement was completed in 60–90 s. The patient was disconnected from the ventilator during 3 s to reach functional residual capacity. Then, 100 ml samples of oxygen were given with a 2 s pause at the end of each inflation until an airway pressure of 60 cmH_2_O was achieved. Finally, 100 ml samples of oxygen were aspirated with a 2 s pause at the end of each deflation until an airway pressure of 0 cmH_2_O was achieved. PV_CF _measurement was preceded by an expiratory pause of 20 s and was completed in 8 s. Ventilatory parameters were set on zero end-expiratory pressure, a respiratory rate of 3 breaths/minute and a tidal volume of 1200 ml to obtain a constant flow of 9 l/minute, thus generating a PV curve on the screen of the ventilator [2]. The maximal peak airway pressure was set at 60 cmH_2_O. When a cycle at low flow was obtained, parameters of the ventilator were immediately set as initially. During the control period, patients were not disconnected from the ventilator and PEEP was unchanged.

All patients had an arterial catheter placed for monitoring of systemic pressures. Blood gases were recorded each minute via a continuous arterial sensor system (Paratrend 7; Diametrics Medical, St Paul, MN, USA) [7]. The 90% response time for the sensor is 180 s or less at 37°C [8]. In humans, the bias provided by the Paratrend 7 was found to be -1.19% for Pao_2 _and +1.28 mmHg for Paco_2 _[7]. During PV_SS_, inspiratory and expiratory flows were measured using a pneumotachograph (Hans-Rudolf 3700; Hans-Rudolf, Kansas City, KS, USA) and a differential pressure transducer. Airway pressures were measured using another differential pressure transducer. Volume changes were obtained by integration of the flow signal recorded using the MP100 data acquisition system (Biopac Systems, Goleta, CA, USA). A static PV curve was constructed to determinate the lower inflection point (LIP) [9] and the upper inflection point (UIP) [10]. The Chord compliance of the respiratory system (Crs) was defined as the slope of the linear part of the PV curve obtained with the super syringe technique.

Variables were expressed as mean ± standard deviation. A two-way analysis of variance (ANOVA) for repeated measures was conducted to study the effects of time and PV measurement on recorded parameters. Positive or negative responders to PV measurement were patients who exhibited an increase or a decrease in Pao_2_/Fio_2 _above 20% occurring in the 5 minutes after PV measurement and persisting for at least 15 minutes. Correlations were analyzed using Pearson product correlation. The maximal increase in Pao_2 _after PV measurement taken using both methods was compared between patients with diffuse, lobar, or patchy ARDS using one-way ANOVA. *P *< 0.05 was considered statistically significant.

## Results

Characteristics of the 17 patients are summarized in Table 1. The Lung Injury Score was 3.1 ± 0.4 and the intensive care unit mortality rate was 36%. Pulmonary infiltrates were classified as diffuse in 11 patients, lobar in three and patchy in three. Tidal volume was 410 ± 96 ml (6.9 ± 1.0 ml/kg of ideal body weight) with a mean inspiratory:expiratory ratio of 1:1.9. All patients had stable haemodynamic parameters (mean arterial pressure [MAP] 76 ± 17 mmHg, heart rate 110 ± 17 beats/minute). Eight patients received norepinephrine (0.2 ± 0.1 μg/kg per minute).

The insufflated volumes were 1824 ± 381 ml (range: 800–2000 ml) during PV_SS _and 1120 ± 115 ml (range: 820–1200 ml) during PV_CF_. The Pplat was 46 ± 9 cmH_2_O at the end of PV_SS _and was 46 ± 11 cmH_2_O at the end of PV_CF_. Peak airway pressure, Pplat, mean airway pressure and Crs (measured 5 minute after PV measurement) were not significantly modified after PV_SS _(36 ± 8 cmH_2_O, 27 ± 7 cmH_2_O, 17 ± 4 cmH_2_O and 58 ± 25 ml/cmH_2_O, respectively) and after PV_CF _(36 ± 6 cmH_2_O, 28 ± 7 cmH_2_O, 18 ± 4 cmH_2_O and 56 ± 29 cmH_2_O, respectively) as compared with baseline values (36 ± 6 cmH_2_O, 27 ± 7 cmH_2_O, 18 ± 4 cmH_2_O and 56 ± 26 ml/cmH_2_O, respectively).

**Figure 1:**
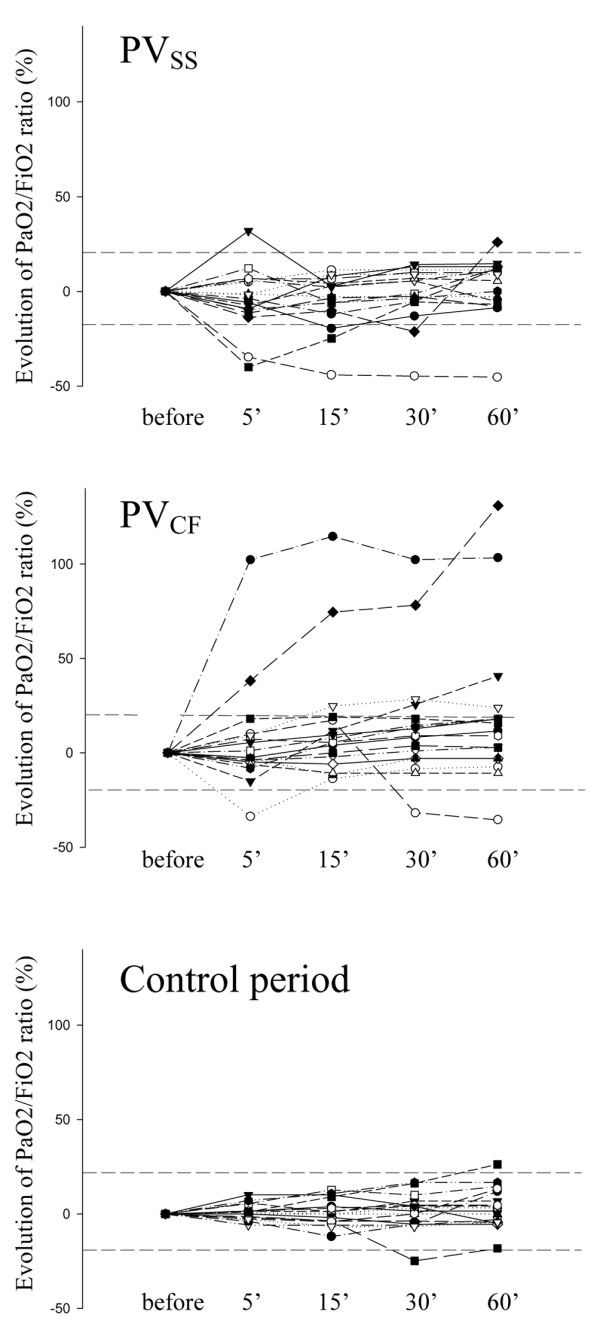
Evolution over time of Pao_2_/Fio_2 _ratio following PV measurements and during a control period. The PV measurements were taken using the super syringe (PV_SS_) and constant flow method (PV_CF_). Data are expressed as percentage increase or decrease in Pao_2_/Fio_2 _ratio at 5, 15, 30 and 60 minutes as compared with values before PV measurement or the control period. Dashed lines represent 20% increase and decrease as compared with before PV measurement. Fio_2_, fractional inspired oxygen; Pao_2_, partial arterial oxygen tension; PV, pressure-volume.

ANOVA revealed that neither PV measurement nor time significantly affected Pao_2 _when measured each minute (*P *= 0.6 for PV measurement; *P *= 0.25 for time; P = 0.2 for interaction). Two patients (patients 7 and 13; Table 1) were positive responders to PV_CF _(Pao_2_/Fio_2 _ratio increased after PV_CF _by 102% in one patient and by 38% in the other; Figure 1). In one patient, Pao_2 _returned to baseline within 2 hours (PV_CF _was followed by control period) whereas the other remained a responder after 3 hours (PV_CF _was the last period in this patient). Two patients were negative responders to PV_SS _(Pao_2_/Fio_2 _ratio decreased by 40% in one patient and by 35% in the other; Figure 1). One patient remained a negative responder 60 minutes after PV_SS _measurement. Neither the Crs nor the Pplat reached during PV measurement was correlated with the maximal increase in Pao_2 _after PV measurement using both methods (data not shown). The maximal increase in Pao_2 _after PV measurement using both methods was not different between patients with diffuse, lobar, or patchy ARDS (data not shown).

Eleven patients exhibited a LIP on the PV curve obtained using the super syringe (Table 1). The PEEP level was 2 cmH_2_O above the LIP on inclusion in one positive responder to PV_CF_, whereas no LIP was identified in the other positive responder. Seven patients exhibited an UIP (at a volume of 1542 ± 82 ml and a pressure of 39 ± 12 cmH_2_O). An UIP was present in the two patients exhibiting a negative response to PV_SS_.

PV measurement did not significantly affect Paco_2 _and MAP. One patient had a decrease in MAP by more than 10 mmHg for less than 5 minutes after PV_SS _and one patient after PV_CF_. After PV_SS_, Paco_2 _increased by more than 10 mmHg in two patients and returned to baseline values after 15 minutes. No case of barotrauma was observed on the chest radiograph performed on the day after the protocol.

## Discussion

Taking the measurements necessary to construct a single PV curve without changing the PEEP level, either by super syringe or by constant flow method, does not durably affect gas exchange and haemodynamic parameters in a population of ARDS patients. Therefore, early evaluation of the impacts of changing ventilator settings or therapeutic interventions should not be influenced by any lasting effect of PV measurement. However, a very limited number of patients exhibit a sustained alteration in oxygenation following PV measurements. Therefore, if a small sample of patients or animals is studied, then a blood gas analysis should be performed before and after PV measurement before any therapeutic intervention is applied, in order to ensure that blood gas analysis is reliable.

Our study compared the two methods commonly used for PV measurement. PV measurement using the constant flow method was able to improve oxygenation over several hours in two of our 17 patients, whereas PV measurement using super syringe impaired Pao_2 _in two patients. This selective effect could be explained by the differences in the design of these PV curve methods. PV measurement using the super syringe consists of a significant phase of alveolar recruitment at inflation but this is followed by an active expiration and by a short disconnection from the ventilator that probably prevents any sustained recruitment. Moreover, this active expiration followed by disconnection could have resulted in a dramatic decrease in Pao_2_, although no such effect was observed in the present investigation. In a recent study Lee and coworkers [3] found that PV measurement using a super syringe was well tolerated in most ARDS patients but caused significant changes in pulse oximetry. However, this latter study did not evaluate for how long oxygenation may be affected by PV measurement. During PV measurement using the constant flow method, the removal of PEEP just before PV curve assessment probably contributed to preventing any significant beneficial effect on oxygenation. The improvement in oxygenation that we observed in two patients could be accounted for by the lack of disconnection from the ventilator and the lack of active expiration as compared with the super syringe procedure. Therefore, PV measurement using the constant flow method could result in significant recruitment in a limited number of ARDS patients.

In the present study, a single PV curve measurement did not affect oxygenation while maintaining a PEEP level of 10 cmH_2_O after PV measurement. Therefore, we cannot rule out the possibility that there is any beneficial influence of increasing PEEP level after PV measurement. Indeed, the effects of a recruitment manoeuvre were suggested to depend on the PEEP level that is applied after that recruitment manoeuvre [11,12]. In our patients, maintaining the PEEP level unchanged after PV measurement might have contributed to an early loss of recruitment possibly achieved during the PV manoeuvre.

We performed only one PV measurement, and therefore we cannot rule out any deleterious effect of PV measurements repeated at short intervals. Indeed, repeated generation of a PV curve using the constant flow method in pigs subjected to lung lavage was recently shown to induce de-recruitment by repeated removal of PEEP [4].

The response to a potential recruitment manoeuvre might depend on the nature of the insult (pulmonary versus extrapulmonary) [13], and on the stage of lung disease (early versus late phase) [14]. Indeed, it is likely that a recruitment manoeuvre is less effective in pulmonary ARDS as well as in late ARDS (for example in patients with more consolidation or fibrosis) [12]. In our study we included mainly patients with pulmonary ARDS. This could have contributed to the lack of beneficial effect of constructing a PV curve on oxygenation. However, our patients presented with early and mainly diffuse ARDS, which should have potentiated the recruitment potentially induced by a PV manoeuvre.

## Conclusion

The effects of a strategy aimed at improving oxygenation can be reliably recorded early after a single PV measurement that is not followed by a change in PEEP level. This finding is important because many clinical and experimental studies report early evaluation findings for therapeutic interventions that are initiated just after PV measurement. Even if a few patients exhibit a sustained improvement in oxygenation (>60 minutes) after PV measurement using the constant flow method, then this latter method cannot be considered a recruitment manoeuvre. We confirmed that PV curve assessment is well tolerated in ARDS patients.

## Key messages

• 	The generation of a single pulmonary PV curve, whether one uses the super syringe or the constant flow method, does not significantly and durably affect oxygenation and haemodynamic parameters in ARDS patients.

• 	Evaluation of the effects of a strategy aiming at improving oxygenation can be reliably recorded early after PV measurement.

## Abbreviations

ANOVA = analysis of variance; ARDS = acute respiratory distress syndrome; Crs = Chord compliance of the total respiratory system; Fio_2 _= fractional inspired oxygen; LIP = lower inflection point; MAP = mean arterial pressure; Paco_2 _= arterial partial carbon dioxide tension; Pao_2 _= arterial partial oxygen tension; PEEP = positive end-expiratory pressure; Pplat = airway plateau pressure; PV = pressure-volume; UIP = upper inflection point.

## Competing interests

The authors declare that they have no competing interests.

## Authors' contributions

AR and LP designed the study and drafted the manuscript. AR performed the statistical analysis. AR, JMF, DD, JMA, SD and MG performed the study. All authors read and approved the final manuscript.

## Figures and Tables

**Table 1:** Characteristics of the patients

Patient	Sex	Age (years)	Diagnosis	SAPS II score	Pao_2_/Fio_2 _ratio (mmHg)	Crs (ml/cmH_2_O)	LIP (cmH_2_O)	Pplat (cmH_2_O)	UIP (cmH_2_O)
1	F	56	Aspiration	42	121	58	15	22	-
2	M	68	CAP	45	95	25	15	40	-
3	M	40	Aspiration	36	89	122	10	19	-
4	M	51	CAP	25	71	34	5	24	54
5	M	31	Aspiration	34	82	43	-	27	-
6	M	41	*Pneumocystis*	36	161	50	12	27	35
7	M	50	CAP	19	110	73	8	24	-
8	M	73	*Legionella*	32	72	75	13	31	-
9	M	31	*Pneumocystis*	9	112	78	-	24	-
10	M	31	Aspiration	74	104	55	7	26	-
11	M	66	Peritonitis	85	55	41	-	32	46
12	F	43	CAP	46	170	75	8	12	27
13	F	42	CAP	22	187	52	-	22	-
14	M	70	Aspiration	40	191	68	-	22	31
15	F	36	CAP	43	216	51	13	27	53
16	F	57	Aspiration	36	172	56	10	30	-
17	F	66	CAP	36	112	12	-	39	26
		50 ± 15		39 ± 18	134 ± 60	56 ± 26		27 ± 7	

Where applicable, results are expressed as mean ± standard deviation. Pao_2_/Fio_2 _ratio is provided under a positive end-expiratory pressure level of 10 cmH_2_O. CAP, community-acquired pneumonia; Crs, Chord compliance of the respiratory system; F, female; Fio_2_, fractional inspired oxygen; LIP, lower inflection point; M, male; Pao_2_, partial arterial oxygen tension; Pplat, plateau airway pressure; SAPS II, Simplified Acute Physiology Score II; UIP, upper inflection point.
